# Molecular Characterization of Three Apple Geminivirus Isolates in Crabapples Detected in Inner Mongolia, China

**DOI:** 10.3390/plants12010195

**Published:** 2023-01-03

**Authors:** Ping-Ping Sun, Lei Zhang, Xiao-Zhao Xu, Mo Zhu, Bin Zhang, Zheng-Nan Li

**Affiliations:** 1 College of Horticulture and Plant Protection, Inner Mongolia Agricultural University, Hohhot 010018, China; 2 College of Horticulture, Qingdao Agricultural University, Qingdao 266109, China; 3 College of Life Sciences, Henan Normal University, Xinxiang 453007, China; 4 College of Life Sciences & Technology, Inner Mongolia Normal University, Hohhot 010028, China

**Keywords:** *Malus asiatica*, Rolling circle amplification, Phylogeny, Recombination, *Apple geminivirus 1*

## Abstract

*Apple geminivirus 1* (AGV) in the genus *Maldovirus* of the family Geminiviridae was first identified infecting apple trees in the year 2015 in China. In this work, we characterized three isolates of the AGV in the Chinese pearleaf crabapple (*Malus asiatica*) in Inner Mongolia Autonomous Region. The viruses were detected by Illumina sequencing and its existence was confirmed by reverse transcription-polymerase chain reaction (RT-PCR) amplification of an AGV fragment. Between the three AGV isolates and the initially characterized AGV isolate PL2015, the nucleotide sequence identities of the complete genome ranged from 91.2 to 91.7%, of the coat protein gene (V1) ranged from 95.4% to 97.3%, and of the replicase gene (C1) ranged from 87.3% to 88.0%. Phylogenetic analysis indicated that the three isolates formed a monophyletic group together with the AGV, separated from the current genera in the family Geminiviridae. This is the first description of the AGV infecting crabapples.

## 1. Introduction

Geminiviruses are plant DNA viruses with a unique twinned particle morphology and monopartite or bipartite single-stranded (ss) circular genomes. Genome components are 2.5–3.6 kb in size. They are grouped in the family Geminiviridae, which currently includes 14 genera (*Becurtovirus*, *Begomovirus*, *Capulavirus*, *Citlodavirus*, *Curtovirus*, *Eragrovirus*, *Grablovirus*, *Maldovirus*, *Mastrevirus*, *Mulcrilevirus*, *Opunvirus*, *Topilevirus*, *Topocuvirus*, and *Turncurtovirus*) [1]. Replication of geminiviruses occur through replicative intermediates of double stranded (ds) DNA by both recombination-dependent and rolling circle mechanisms, and bidirectional dsDNA transcription produces viral mRNAs and translation of viral proteins. It was proved that the entire DNA genome sequences of geminiviruses are densely covered with viral siRNAs (21–24 nt) in both sense and antisense orientations. The complete genomes, therefore, can be reconstructed by deep sequencing and de novo assembly of viral siRNAs using bioinformatics tools [2]. *Apple geminivirus* 1 (AGV) is a member in the family Geminiviridae, belonging to the genus *Maldovirus* [3]. AGV PL2015 (GenBank accession no. KM386645) is the exemplar isolate of the species, and its genome has six genes: V1 and V2 on the virion (v)-sense strand and C1, C2, C3, and C4 on the complementary (c)-sense strand. This genomic organization is similar to that of monopartite begomoviruses, although the nucleotide sequence identities between the AGV and begomoviruses are low, ranging from 63.5% to 66.1% for the full genomes [3]. V1 encodes the coat protein and V2 is a post-transcriptional gene silencing suppressor [4]. The proteins encoded by C1, C2, C3 and C4 are supposed to code for the replicase (Rep), transcriptional activator, replication enhancer and symptom determinant, respectively [3,4].

The Chinese pearleaf crabapple (*Malus asiatica*) is widely grown in north China for its beautiful blossoms in gardens, and an increasing planting area is seen for crabapples for its commercial value. The fruit is usually processed to dried apple slices, fruit flakes, or roll-ups at good market prices. It also has elite germplasm due to its excellent cold hardiness; for example, the rootstock KM, which has superior cold hardiness with resistance to −35 °C, is a cross between *M. asiatica* (the female parent) and M9 (the male parent) [5,6]. Several viruses have been described infecting crabapples in China, such as the capillovirus *Apple stem grooving virus* (ASGV) [7], the trichovirus *Apple chlorotic leaf spot virus* (ACLSV) [8,9], and the ilarvirus *Apple necrotic mosaic virus* [10]. In this work, we reported the detection and genome characterization of the AGV isolates in mixed infection with several RNA viruses in Chinese pearleaf crabapples in Inner Mongolia, China. This was the first record of the AGV infecting crabapples.

## 2. Results

### 2.1. Analysis of Small RNA Sequencing Data and RT-PCR Validation

By NGS, a total of 21,883,344 raw reads were obtained and 19,426,571 clean reads with lengths of 18–35 nt remained after adapters removal. After removing the non-coding RNA and repeated sequences, a total of 11,308,324 clean reads were assembled into 595 contigs with N50 of 62 using Velvet Software.

BLAST alignment found that 18 out of the 595 contigs were mapped to the AGV genome sequences, 6 to the ASGV, 4 to the apple necrotic mosaic virus (ApNMV) and 1 to the hollyhock leaf crumple virus (HoLCrV). RT-PCR products of an expected size of about 270, 110, and 180 bp were amplified for detection of the AGV, ASGV and ApNMV, respectively. The RT-PCR amplification and sequences of the amplicons demonstrated that three samples were positive for AGV infection, eight for the ASGV, and five for the ApNMV; no product of the HoLCrV was generated in RT-PCR amplification (Supplementary Table S1).

### 2.2. RCA Amplification of AGV Genomic DNA

Total DNA was extracted from each of the three samples that were RT-PCR positive for the AGV and used for RCA detection of the circular genomic DNA. After digestion with the restriction enzyme *Bam*HI, products in identical size of about 3 kb were observed for all the three samples (Supplementary Figure S1). The results confirmed that the AGV genome is composed of a single component.

### 2.3. Determination and Characterization of AGV Genome Sequence

Complete genomes of three isolates of the AGV were obtained, named as the AGV isolates Ningcheng (NC)1, NC2, and NC3; the isolate NC1 included two variants, generated from a single sample, marked as NC1a and NC1b. The genome sequences were annotated and submitted to GenBank under accession numbers MT107529 (AGV NC1a), MT107530 (AGV NC1b), MT107531 (AGV NC2), and MT107532 (AGV NC3). The complete genome was of 2946 nt for AGV NC1a, NC1b, and NC2, and of 2945 nt for NC3. They have a larger genome in comparison with AGV PL2015 (KM386645) whose genome consists of 2932 nucleotides. The three isolates shared the same genome organization with AGV PL2015, i.e., V1 and V2 genes on the v-sense strand, encoding proteins of 255 and 98 amino acid residues, respectively; C1, C2, C3 and C4 on the c-sense strand, encoding proteins of 352 (Rep), 166 (or 167 in AGV NC3), 134 and 77 amino acid residues, respectively. The nona-nucleotide sequence of TAATATT↓AC, which is conserved in geminiviruses, were observed for all three isolates. It was shown that the three AGV isolates have longer intergenic regions (IRs) (409 or 412 nt) than PL2015 (398 nt), which was totally responsible for the larger genome size.

Pairwise alignments of the complete genomes showed that the nucleotide sequence identities among the three AGV isolates ranged from 93.9 to 99.8%; NC1a and NC1b have the highest identity of 99.8% (2945 nt / 2950 nt; only mutations at five sites within the C1 genes), while NC2 and NC3 have the lowest identity of 93.9% with each other (Supplementary Table S2). In addition, pairwise alignments of each gene among the three isolates indicated that the C1 genes share the lowest nucleotide sequence identities of 94.1–99.5% with one another, while the C2, C3, C4, V1 and V2 genes have nucleotide sequence identities of 95.6–100%, 98.3–100%, 97.4–100%, 95.7–100%, and 96.3–100%, respectively, with one another (Supplementary Table S2). Pairwise alignments of amino acid sequences were conducted for each protein of AGV as well. Among the four isolates, the amino acid sequence identity was of 86.1–99.4% for the C1 proteins, 90.5–100% for C2 proteins, 95.6–100% for C3 proteins, 93.6–100% for C4 proteins, 98.4–100% for V1 proteins, and 96.6–100% for V2 proteins.

The nucleotide sequence identities between AGV PL2015 and the three isolates were 91.2–91.7%, which was lower compared to those among the three isolates themselves, as was also the case for the identities of each gene and the amino acids (Supplementary Table S2).

### 2.4. Phylogeny between AGV and Other Geminiviruses

**Figure 1:**
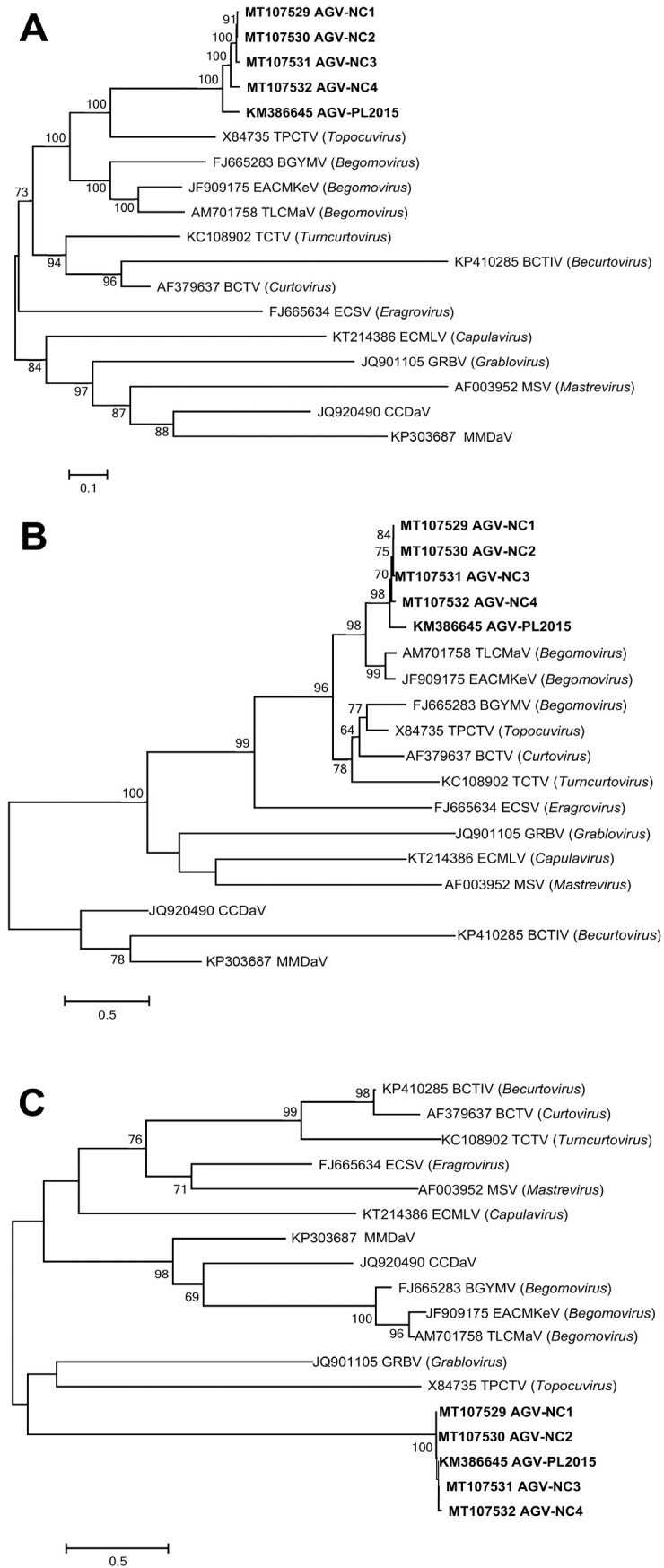
Phylogenetic relationships of AGV and other members in the family Geminiviridae. The trees were reconstructed based on complete genome sequences (**A**), amino acid sequences of Rep (**B**) and amino acid sequences of CP (**C**), respectively. AGV isolates are marked in bold. Numbers on the branches indicate bootstrap percentage after 1000 replications in constructing the tree. Scale bar refers to a phylogenetic distance of 0.5 or 0.1 nucleotide substitutions per site.

On the phylogenetic tree (Figure 1) reconstructed based on the complete genome sequences of the representatives of the family Geminiviridae, AGV NC1a, NC1b, NC2 and NC3 clustered together and were closely related to PL2015, forming a monophyletic group, while the AGV isolates were distinct from other members in the family Geminiviridae. The same results were seen on the trees constructed based on the amino acid sequence of Rep or CP as well (Figure 1).

### 2.5. Recombination Analysis

Using seven different methods in the Recombination Detection Program v.3.44 software, recombination events were predicted for the AGV isolates, i.e., AGV NC3 and PL2015 were as a major and a minor parent; the major parent is the parental sequence that contributes the larger fraction, while the minor parent is the parental sequence that contributes the smaller fraction of the recombinant sequence. The recombination region was seen in nt 1221–2751, resulting in the other two isolates of AGV NC1a, NC1b, and NC2 (Table 1).

## 3. Discussion

RCA in combination with RFLP is a highly reproducible tool for geminivirus diagnosis, largely independent of viral genome organization. In a single reaction, all infecting circular DNA components are amplified in a single step without any knowledge of sequence information, including the genome of distinct species and strains, defective subviral DNA, and presumably the circular satellite DNAs of geminiviruses [11]. The amplified viral DNA can be characterized by restriction fragment length polymorphism analysis. In this work, the AGV contigs were recognized in samples of Chinese pearleaf crabapples using NGS technology. Based on the contigs, specific primers were then designed with which RT-PCR was performed and the existence of the AGV in the crabapples was confirmed. Followed by RCA/RFLP technique, the AGV isolates were proved to be monopartite. AGV infection was finally determined in the symptomatic leaf samples of Chinese pearleaf crabapples. The infection of the ASGV and ApNMV, however, were detected in the samples as well, indicating the mixed infection in the samples. The disease symptoms on the crabapples, therefore, cannot be completely attributed to the infection of the AGV, and the role of the AGV in the symptom determinant is not clear yet. In the first publication on the AGV, the infection of AGV PL2015 on *Nicotiana benthamiana*, *N. glutinosa* and *Solanum lycopersicum* was confirmed to be symptomless using the biologically active clone of the complete genome of PL2015 [3], suggesting the symptomless AGV infection. A functional scanning of AGV PL2015 proteins, however, indicated that V2, C1, and C4 are important symptom determinants on *N. benthamiana*, inducing severe symptoms such as crinkling, necrosis, and upward leaf curling [4]. The damage of the AGV on *Malus* sp. is unknown and needs further study.

Geminiviruses possess high genetic variability due to their high nucleotide substitution rates [12,13] and frequent occurrence of recombination [14]. In this work, the lowest nucleotide sequence identity of the C1 genes (encoding Rep) was seen for the five currently available AGV isolates, indicating a higher genetic variability of the C1 gene than the other five genes of the AGV, which corresponds closely to other members in the family Geminiviridae. For example, cotton leaf curl geminivirus (CLCuV) has a very high genetic variability in Rep and CP [15]. In addition, recombination is a pervasive process generating diversity in most viruses, joining variants that arise independently within the same molecule. It has often been associated with host switches and host range expansion, creating new opportunities for viruses to overcome selective pressures [16]. Studies on recombination provide important evolutionary information on host ranges and virus emergence [17]. In this work, recombination events were predicted for the five AGV isolates as well and AGV NC3 and PL2015 were a major and a minor parent. Although mechanisms of the recombination are less clear, it is claimed that geminiviruses are rapidly diversifying owing to the transportation of plant materials, new agricultural practices, global trade, climate change, and viral evolution [18]. In fact, AGV PL2015 was detected in Qixia City, Shandong province of China, which is geographically close to Ningcheng where the AGV NC1a, NC1b, NC2 and NC3 were detected, with an air-line distance of about 475 km, crossing over the Bohai Sea. Shandong province is a main area for apple production in the Bohai Gulf region of China [19], producing a large portion of apple fruit and commercial seedlings in China. Possibly the AGV spread has accompanied the apple seedling trade among different regions in China, which might explain the suggested recombination between AGV NC3 and PL2015.

In addition, IRs of geminiviruses were reported harboring important elements for viral functions, such as viral transcription enhancer elements [20]. AGV NC1a, NC1b, NC2, and NC3 in the crabapple have a longer IR than the PL2015 in “Fuji” apple, with the main motif of AGGTGGGA. However, in comparison with the published transcription enhancer elements, a rarely conserved sequence was observed. The role of the longer IR in the AGV isolates was unknown; insufficient data were available on the AGV to elucidate the new viral pathogen. This was the first description of AGV on crabapples, and much more research is needed to address the virus disease.

## 4. Materials and Methods

### 4.1. Source of Virus

In July 2018, Chinese pearleaf crabapple trees showing symptoms of mosaic and yellowing, indicative of virus infection, were observed in Ningcheng County, Inner Mongolia of China. Fresh leaf samples were randomly collected from nine symptomatic trees. All samples were frozen in liquid nitrogen immediately and transferred and stored at −86 °C for further use.

### 4.2. Total RNA Extraction, Small RNA Sequencing and Validation of Candidate Viruses

In order to identify viruses in the samples, total RNA was extracted from pooled leaf samples using TRIzol reagent (Invitrogen) according to the manufacturer’s instructions. The extracted RNA was then used for constructing a small RNA library as described by Mi et al. [21] and was sequenced via next-generation sequencing (NGS) using Illumina HiSeq2000 platform at Biomarker Technologies (Beijing, China). The obtained raw reads were cleaned by trimming adapter sequences and eliminating reads less than 18 nt or more than 35 nt. The non-coding RNAs and repeated sequences were filtered out from the clean reads using Bowtie software v.1.2.3 [22], and the remaining reads were assembled de novo into contigs using Velvet Software v.1.2.10 [23]. The contigs underwent BLAST search for homologous sequences in the GenBank Virus RefSeq Nucleotide and Virus RefSeq Protein databases, with e-values of 10^−5^ [24].

To confirm the occurrence of NGS-detected viruses in the collected samples, RT-PCR was performed to amplify viral fragments using designed primer pairs: AGV-detF: GTCGTCAAGCTCGTCCAGAT/AGV-detR: GAAGAAGGAGAGTCAACTGCG, ASGV-detF: GCTGTTACTTTGGACTCAGACC/ASGV-detR:TATCCTGTTCATACTGTGGGCA, ApNMV-detF: CACGATAGAGTCCTGGCGAG/ApNMV-detR: CCTGGAAGAACCTGTCATCG, HoLCrV-detF: CTTACTTCCTGAGTTAAGCGC/HoLCrV-detR: GGTATCCCCAAGCAGATCAACAC. Total RNA of each of the nine samples was extracted as described above; the primers were designed based on the obtained viral contigs. Single-stranded cDNA was synthesized from the total RNA using Murine MLV-reverse transcriptase (Promega, Beijing, China) with random hexamer primer according to the manufacturer’s instructions. The PCR assays were set up in a volume of 25 μL containing 1.0 μL cDNA, 12.5 μL Premix LA Taq DNA polymerase (TaKaRa, San Jose, CA, USA), 1.0 μL 10 μM forward and reverse primers, with 35 cycles of 94 °C for 30 s, 55 °C for 30 s and 72 °C for 1 min. The PCR products were gel-purified using a Gel Extraction Kit (CWBio, Beijing, China) for sequencing.

### 4.3. Total DNA Extraction and Rolling Circle Amplification (RCA)

Total DNA was extracted from each of the AGV-positive samples using Easy Pure Plant Genomic DNA Kit (TransGen, Beijing, China) following the manufacturer’s instructions. The extracted DNA was used as template in RCA, which was performed using Illustra TempliPhi 100 Amplification kit (GE Healthcare, Chicago, IL, USA) according to the manufacturer’s instructions. The RCA products were digested with the restriction enzyme *Bam*HI and separated on 1% agarose by electrophoresis.

### 4.4. Determination of Full-Length AGV Genome and Sequence Analysis

To amplify the complete genome of AGV, PCR assay was carried out as described above, except that primer pair of AGV-F: CGTGTATTGGGTTCTTCAGAC/AGV-R: TGAGCATCGACCTCATTCGG was applied to the PCR amplification and the total DNA above substituted for cDNA as template. The primers were designed based on the sequence flanked by primers AGV-detF/AGV-detR. The PCR products were gel-purified using Gel Extraction Kit (CWBIO, Beijing, China), cloned into pMD18-T simple vector (TaKaRa, USA), and *Escherichia coli* JM109 competent cells were transformed. Selected clones (three per amplicon) were sequenced at GENEWIZ, Inc., Beijing, China.

The full-length genome sequences were assembled using Vector NTI (Invitrogen) based on overlapping regions, and the ORFs were identified using ORFfinder (https://www.ncbi.nlm.nih.gov/orffinder, accessed on 15 May 2022). SDT 1.0 [25] was used to determine pairwise nucleotide sequence identities and amino acid sequences among different isolates.

### 4.5. Phylogenetic Analysis

Phylogeny of AGV and other members in the family Geminiviridae were analyzed using MEGA6.0 software [26]. Representatives of 11 genera in the family *Geminiviridae*, and of the one unassigned species, were selected as references, including Beet curly top Iran virus (BCTIV, KP410285), Bean golden mosaic virus (BGYMV, FJ665283), East African cassava mosaic virus-Kenya isolate, (EACMKeV, JF909175), Tomato leaf curl Anjouan virus (TLCMaV, AM701758), Euphorbia caput-medusae latent virus (ECMLV, KT214386), Beet curly top virus (BCTV, AF379637), Eragrostis curvula streak virus (ECSV, FJ665634), Grapevine red blotch virus (GRBV, JQ901105), Maize streak virus (MSV, AF003952), Tomato pseudo-curly top virus (TPCTV, X84735), Turnip curly top virus (TCTV, KC108902), Citrus chlorotic dwarf associated virus (CCDaV, JQ920490), and Mulberry mosaic dwarf associated virus (MMDaV, KP303687). Phylogenetic trees were reconstructed based on the nucleotide sequences of complete genome, amino acid sequences of Rep, and amino acid sequences of CP, respectively. The sequences were retrieved from NCBI GenBank database. To construct a tree, sequences were aligned using ClustalW and built up by neighbor-joining method with bootstrapping of 1000 replicates. Only bootstrap values above 60 are shown on the tree.

### 4.6. Recombination Analysis

Recombination analysis was performed among the AGV isolates using seven different methods in Recombination Detection Program v.3.44 [27], including RDP, GENECONV, CHIMERA, BOOTSCAN, MAXCHI, SISCAN, and 3SEQ. Only recombination events predicted by at least four methods with *p*-value < 0.01 were considered.

## Figures and Tables

**Table 1:** Recombination events among the five apple geminivirus isolates predicted by RDP.

Recombinant	Parental Isolate		Region (nt)	*p*-Value ^a^						
	Major	Minor		R	G	B	M	C	S	3S
AGV-NC1a	AGV-NC3	AGV-PL2015	2751-1221	4.3 × 10^−3^	-	5.4 × 10^−3^	5.6 × 10^−7^	2.4 × 10^−9^	-	-
AGV-NC1b	AGV-NC3	AGV-PL2015	2751-1221	4.3 × 10^−3^	-	5.4 × 10^−3^	5.6 × 10^−7^	2.4 × 10^−9^	-	-
AGV-NC2	AGV-NC3	AGV-PL2015	2751-1221	4.3 × 10^−3^	-	5.4 × 10^−3^	5.6 × 10^−7^	2.4 × 10^−9^	-	-

^a^*p*-values for each recombination event detected by methods: R, RDP; G, GENECONV; B, BOOTSCAN; M, MAXCHI; C, CHIMAERA; S, SISCAN; 3S, 3SEQ.

## Data Availability

The data presented in this study are openly available in GenBank, under accession numbers MT107529 (AGV NC1a), MT107530 (AGV NC1b), MT107531 (AGV NC2), and MT107532 (AGV NC3).

## Supplementary Materials

The following supporting information can be downloaded at: https://www.mdpi.com/article/10.3390/plants12010195/s1, Figure S1: The monopartite genome fragments of AGV. The fragments were produced by digestion of RCA products of AGV with restriction enzyme *Bam*HI. M: AL10000 DNA Marker (BLKW Inc., Beijing, China), from top to bottom the fragment size was 10, 6, 4, 3, 2, and 1 kp; 1–3: apple geminivirus-infected apple samples; Table S1: Detection of apple geminivirus (AGV), apple stem grooving virus (ASGV), apple necrotic mosaic virus (ApNMV) and hollyhock leaf crumple virus (HoLCrV) by RT-PCR from the nine samples, Table S2: Pair distances of each gene of the five AGV isolates. Left: nucleotide sequence identities; right: amino acid sequence identities.
