# Supplementary material for: Molecular Characterization of Three Apple Geminivirus Isolates in Crabapples Detected in Inner Mongolia, China
Source: Plants (Basel). 2023 Jan 3;12(1):195. doi: 10.3390/plants12010195 (PMC9824349; doi:10.3390/plants12010195)
Supplement: Supplementary file 1 [file plants-12-00195-s001.zip › plants-2108504-supplementary.pdf]

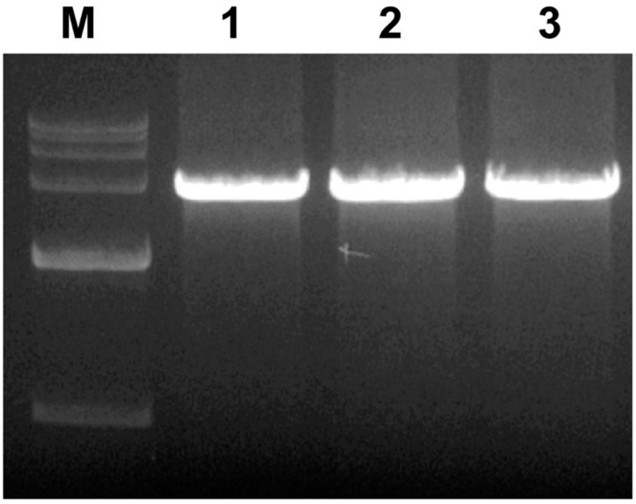

Figure S1. The monopartite genome fragments of AGV. The fragments were produced by digestion of RCA products of AGV with restriction enzyme *Bam*HI. M: AL10000 DNA Marker (BLKW Inc., Beijing, China), from top to bottom the fragment size was 10, 6, 4, 3, 2, and 1 kp; 1–3: apple geminivirus-infected apple samples.

Table S1. Detection of apple geminivirus (AGV), apple stem grooving virus (ASGV), apple necrotic mosaic virus (ApNMV) and hollyhock leaf crumple virus (HoLCrV) by RT-PCR from the nine samples.

Sample	RT-PCR detection			
	AGV	ASGV	ApNMV	HoLCrV
1	+ (NC1a, NC1b)	+	-	-
2	-	+	+	-
3	+ (NC2)	+	+	-
4	+ (NC3)	+	-	-
5	-	-	-	-
6	-	+	+	-
7	-	+	+	-
8	-	+	+	-
9	-	+	-	-

Table S2. Pair distances of each gene of the five AGV isolates. Left: nucleotide sequence identities; right: amino acid sequence identities.

AGV		NC3	NC2	NC1b	NC1a					
	PL2015	91.2	91.5	91.5	91.7					
	NC1a	94.1	98.8	99.8						
	NC1b	94.2	98.9							
	NC2	93.9								
C1		NC3	NC2	NC1b	NC1a		NC3	NC2	NC1b	NC1a
	PL2015	88.0	87.3	87.4	87.9	PL2015	75.6	74.5	75.1	75.6
	NC1a	94.2	98.7	99.5		NC1a	87.0	98.0	99.4	
	NC1b	94.5	99.2			NC1b	87.0	98.6		
	NC2	94.1				NC2	86.1			
C2		NC3	NC2	NC1b	NC1a		NC3	NC2	NC1b	NC1a
	PL2015	94.2	95.2	94.4	94.4	PL2015	86.9	90.5	88.1	88.1
	NC1a	96.0	99.2	100.0		NC1a	91.7	97.6	100.0	

	NC1b	96.0	99.2			NC1b	91.7	97.6		
	NC2	95.6				NC2	90.5			
C3		NC3	NC2	NC1b	NC1a		NC3	NC2	NC1b	NC1a
	PL2015	96.3	95.1	95.8	95.8	PL2015	93.3	90.4	92.6	92.6
	NC1a	99.0	99.3	100.0		NC1a	97.8	97.8	100.0	
	NC1b	99.0	99.3			NC1b	97.8	97.8		
	NC2	98.3				NC2	95.6			
C4		NC3	NC2	NC1b	NC1a		NC3	NC2	NC1b	NC1a
	PL2015	94.9	94.4	94.4	94.4	PL2015	85.9	84.6	84.6	84.6
	NC1a	97.4	100.0	100.0		NC1a	93.6	100.0	100.0	
	NC1b	97.4	100.0			NC1b	93.6	100.0		
	NC2	97.4				NC2	93.6			
V1		NC3	NC2	NC1b	NC1a		NC3	NC2	NC1b	NC1a
	PL2015	95.4	97.0	97.3	97.3	PL2015	98.4	100.0	99.6	99.6
	NC1a	96.1	98.7	100.0		NC1a	98.8	99.6	100.0	
	NC1b	96.1	98.7			NC1b	98.8	99.6		
	NC2	95.7				NC2	98.4			
V2		NC3	NC2	NC1b	NC1a		NC3	NC2	NC1b	NC1a
	PL2015	96.3	98.0	98.3	98.3	PL2015	96.3	98.0	98.3	98.3
	NC1a	96.3	99.0	100.0		NC1a	96.3	99.0	100.0	
	NC1b	96.3	99.0			NC1b	96.3	99.0		
	NC2	96.6				NC2	96.6			
